# Long-term Persistence of *Opisthorchis viverrini* Antigen in Urine: A Prospective Study in Northeast Thailand

**DOI:** 10.4269/ajtmh.22-0478

**Published:** 2022-12-26

**Authors:** Chanika Worasith, Phattharaphon Wongphutorn, Kulthida Y. Kopolrat, Chutima Homwong, Anchalee Techasen, Raynoo Thanan, Chompunoot Wangboon, Chatanun Eamudomkarn, Jiraporn Sithithaworn, Thomas Crellen, Paiboon Sithithaworn

**Affiliations:** ^1^ Department of Parasitology, Faculty of Medicine, Khon Kaen University, Thailand;; ^2^ Cholangiocarcinoma Research Institute, Khon Kaen University, Khon Kaen, Thailand;; ^3^ Biomedical Science Program, Graduate School, Khon Kaen University, Khon Kaen, Thailand;; ^4^ Faculty of Public Health, Kasetsart University Chalermphrakiat Sakon Nakhon Province Campus, Sakon Nakhon, Thailand;; ^5^ Faculty of Associated Medical Sciences, Khon Kaen University, Khon Kaen, Thailand;; ^6^ Department of Biochemistry, Faculty of Medicine, Khon Kaen University, Khon Kaen, Thailand;; ^7^ School of Preclinical Science, Institute of Science, Suranaree University of Technology, Nakhon Rachasima, Thailand;; ^8^ School of Biodiversity, One Health and Veterinary Medicine, Glasgow, United Kingdom;; ^9^ Big Data Institute, Nuffield Department of Medicine, University of Oxford, Oxford, United Kingdom

## Abstract

Antigen detected in urine for the diagnosis of opisthorchiasis has a low daily variation; however, the longer term variability in antigen concentrations is unknown. In this study, we prospectively monitored *Opisthorchis viverrini* antigen concentrations for 30 consecutive days and at subsequent monthly intervals in a cohort of opisthorchiasis-positive individuals. On the basis of the monoclonal antibody–based ELISA, the profiles of antigen-positive rate and antigen concentration exhibited no significant change over 30 days with a mean proportion positive of 87.1% (range 73.7%–100%), and the average antigen concentration was 29.7 ± 2.2 ng/mL (mean ± SE). The urine antigen concentration at baseline was similar to the subsequent measurements at 2, 4, 6, and 10 months in the follow-up study (*P* > 0.05). The consistency and low daily and long-term fluctuation of *O. viverrini* antigen in urine demonstrates the reliability of urine assay for diagnosis of opisthorchiasis.

The liver fluke, *Opisthorchis viverrini*, is the causative agent of opisthorchiasis and is a known risk factor for cholangiocarcinoma (CCA) in Southeast Asia including Thailand, the Lao People’s Democratic Republic, Cambodia, and Vietnam.^1^^,^^2^ A comprehensive control program and elimination of the liver fluke is an important strategy for reduction of CCA.^3^^,^^4^ Diagnosis of *O. viverrini* infection by fecal examination has limited sensitivity, which can only be overcome by examination over multiple days; however, this increases the cost and logistical complexity of diagnostic surveys.^5^ Alternative diagnostic methods for *O. viverrini* include antigen detection by copro-antigen^6^ and urine antigen detection,^7^^,^^8^ which have been shown to have a higher sensitivity than fecal egg examination. These antigen assays offer high diagnostic performance, particularly in individuals with low worm burden and/or undetectable number of eggs in feces. A community-based survey using the urine antigen assay demonstrated a correlation in the prevalence and intensity of infection with fecal egg examination.^7^^,^^8^ In addition to screening and diagnosis of opisthorchiasis, the urine assay has been used for monitoring outcomes of drug treatment and reinfection.^9^ Although the urine assay has several advantages over fecal egg examination, past studies have relied on a single urine sample for diagnosis. We recently reported low and insignificant daily variation in the level of urine antigen over three days, which strengthens the evidence base for the reliability of urine assay for detecting opisthorchiasis. However, data on the variation of antigen in urine over extended periods has not been investigated.

In this study, we evaluated the variation in *O. viverrini* antigen levels over 30 consecutive days in addition to the longer term variation, considering both qualitative (proportion positive) and quantitative (antigen concentration) diagnosis of opisthorchiasis in two cohorts of participants (age > 15 years) in the subdistrict Muang within Khon Kaen province, Northeast Thailand. The first morning urine samples (10 mL) were collected daily from the participants for urine assay. On day 0 and day 30, fecal samples (10 g) were collected for parasite examination by quantitative formalin-ethyl acetate concentration technique (FECT). The protocol for *O. viverrini* antigen detection in urine has been described previously.^7^^,^^8^ Briefly, the monoclonal antibody- based ELISA uses a monoclonal antibody and IgG rabbit anti *O. viverrini* antigen. At the end of the procedure, the plates were read using a plate reader (Tecan Sunrise Absorbance Reader, Salzburg, Austria) at 492 nm to calculate the antigen concentration by the standard curve.^7^^,^^8^ A sample was considered positive for *O. viverrini* when the antigen was > 19.4 ng/mL. In this study, the human experimental protocol was approved by the Khon Kaen University Ethics Committee (HE561478). Informed consent was obtained from all subjects, and those with *O. viverini* eggs positive by FECT and/or urine antigen assay were treated with a single oral dose of praziquantel (40 mg/kg body weight).

The participants (*N* = 75) were first screened for *O. viverrini* infection, and the antigen-positive participants (*n* = 42) consisted of 22 males and 20 females aged between 22 and 87 years (mean ± SD, 55.4 ± 13.8). The participants provided urine samples for 24 to 30 consecutive days. The percentage *O. viverrini* positive ranged between 73.7% and 100% per day with the mean percentage positive on a single day being 87.1%. The antigen levels (nanogram/milliliter) showed no significant fluctuation between days (repeated-measures analysis of variance, *F* = 39.98, *P* > 0.05) with the mean values between 29.7 ± 2.2 ng/mL (Figure 1). FECT showed a negative result for all participants (*N* = 42) screened by fecal examination (day 0), and five participants (11.9%) were positive for *O. viverrini* eggs on day 30 with fecal egg counts from six to 18 eggs per gram.

**Figure 1.**
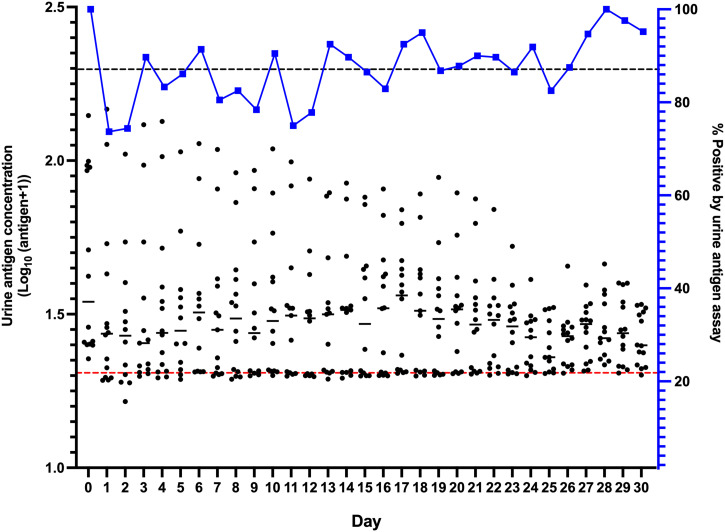
The profiles of positive detection rate for opisthorchiasis and urine antigen concentration (nanograms per milliliter) over 30 days. The percentage urine antigen positive in each day (x-axis) correspond to the right y-axis (%) with an overall prevalence of 87.1 (black-dotted line). Data shown (dots) are urine antigen concentrations for individual participants, which correspond to the left y-axis (log-transformed antigen concentration, nanograms per milliliter). The red-dotted line indicates the cutoff value for a positive urine antigen assay (> 19.4 ng/mL). Urine samples were obtained from 35 to 42 participants per day.

To observe variability of urine antigen levels over a longer duration, another cohort of urine-positive participants for opisthorchiasis (*N* = 57) consisted of 18 males and 39 females with mean age 57.8 years (SD 11.5 years). The participants were asked to donate urine samples at baseline at the beginning of the experiment and provided subsequent samples at 2 months (*N* = 21), 4 months (*N* = 14), 6 months (*N* = 11), and 10 months (*N* = 11), respectively. Comparisons of antigen concentrations between baseline (month 0) and subsequent samplings at months 2, 4, 6, and 10 showed no significant difference (Mann–Whitney *U* test, *P* > 0.05) (Table 1).

**Table 1:** The concentrations of antigen in urine (nanograms per milliliter) in opisthorchiasis participants measured by monoclonal antibody–based ELISA at baseline compared with follow up intervals of 2, 4, 6, and 10 months

Follow-up interval (months)	No. assessed	Antigen concentration, mean ± SD, (ng/mL), initial/follow up	*P* value*
2	21	31.2 ± 6.8 / 33.6 ± 6.0	0.182
4	14	40.7 ± 14.7 / 36.3 ± 11.3	0.427
6	11	30.2 ± 11.6 / 33.6 ± 14.6	0.847
10	11	38.5 ± 20.7 / 36.4 ± 13.7	0.948

* Mann–Whitney *U* test.

On the basis of these results, which show persistent qualitative and quantitative antigen detection in urine, we argue that this will help resolve the limitations of fecal egg examination. The results showed negligible variation of antigen in urine when determined daily for 30 days or at an interval of 10 months. Antigen detection in urine samples provides advantages not only for diagnosis of light infection but also early stage (prepatent) infections where the worms are still in the immature stage and have not reached full reproductive age.^10^ Our study supports this possibility because five of 42 individuals (11.9%) had six to 18 eggs per gram of *O. viverrini* on day 30 of the study. Possible explanations included low worm burden or a small number of very old, degenerating worms. Another explanation for the low sensitivity of FECT was that fecal examination was not performed during days 1 through 29 and also a single fecal sample examination was used, whereas repeated fecal examinations can improve the diagnosis.^5^ The fluctuation of antigen in urine, where participants who originally had positive urine antigen tests but tested negative in subsequent days, was observed in 15% of individuals. This particular group of people had the antigen levels fell within 10% above the cutoff level and whether it associated with the dilution effects of antigen in urine due to water intake or not required more study.^11^

We previously showed that a positive diagnosis with the urine antigen diagnostic indicates active or current *O. viverrini* infection because the antigen levels diminished after curative treatment in either fecal egg positive and negative individuals^9^ and antigen reemerged because of reinfection by *O. viverrini* 40 weeks later. Evidence from animal models also indicates that the antigen detected in urine is directly linked to the presence of adult worms in the liver.^10^ Therefore, human and animal studies support the reliability of urine antigen detection to indicate active or current opisthorchiasis.

In conclusion, the results showed a low variation of *O. viverrini* antigen levels in urine over 30 consecutive days and also over a longer interval of 2 to 10 months. Furthermore, the findings of antigen positivity in urine in *O. viverrini* egg negative individuals may represent possible prepatent infection, low worm burden, or old-age worms. Unlike fecal examination, a single urine sample examination for *O. viverrini* antigen gave a consistent diagnosis, which lends further support for the usefulness of urine antigen assay in screening for opisthorchiasis. Given the greater social acceptability of urine sample collection compared with fecal collection, further development of the antigen detection method into a rapid point of care test platform is being undertaken to facilitate the screening and control of opisthorchiasis.
